# Laser-Assisted Printed Flexible Sensors: A Review

**DOI:** 10.3390/s19061462

**Published:** 2019-03-25

**Authors:** Tao Han, Anindya Nag, Nasrin Afsarimanesh, Subhas Chandra Mukhopadhyay, Sudip Kundu, Yongzhao Xu

**Affiliations:** 1 DGUT-CNAM Institute, Dongguan University of Technology, Dongguan 523106, China; hant@dgut.edu.cn (T.H.); xuyz@dgut.edu.cn (Y.X.); 2 School of Engineering, Macquarie University, Sydney 2109, Australia; afsarimanesh.n@gmail.com (N.A.); subhas.mukhopadhyay@mq.edu.au (S.C.M.); 3 CSIR-Central Mechanical Engineering Research Institute Durgapur, West Bengal 713209, India; sudip.skundu.94@gmail.com

**Keywords:** printed, nanocomposite, laser induced, graphene, carbon nanotubes, 3D-printed

## Abstract

This paper provides a substantial review of some of the significant research done on the fabrication and implementation of laser-assisted printed flexible sensors. In recent times, using laser cutting to develop printed flexible sensors has become a popular technique due to advantages such as the low cost of production, easy sample preparation, the ability to process a range of raw materials, and its usability for different functionalities. Different kinds of laser cutters are now available that work on samples very precisely via the available laser parameters. Thus, laser-cutting techniques provide huge scope for the development of prototypes with a varied range of sizes and dimensions. Meanwhile, researchers have been constantly working on the types of materials that can be processed, individually or in conjugation with one another, to form samples for laser-ablation. Some of the laser-printed techniques that are commonly considered for fabricating flexible sensors, which are discussed in this paper, include nanocomposite-based, laser-ablated, and 3D-printing. The developed sensors have been used for a range of applications, such as electrochemical and strain-sensing purposes. The challenges faced by the current printed flexible sensors, along with a market survey, are also outlined in this paper.

## 1. Introduction

The introduction of sensing technology for monitoring purposes has improved the quality of human life. Nowadays, sensors are utilized ubiquitously for different day-to-day applications, including in health [1,2], domestic [3,4], and industrial [5,6] settings. After the introduction of semiconducting sensors around two decades ago [7,8], silicon sensors became highly popular due to their high sensitivity and low-contact resistances. Nonetheless, there were certain disadvantages with these, such as their high fabrication cost, high input power, and low robustness, which led researchers to seek alternative options. As a complement to rigid sensors, flexible sensors [9,10,11] became a popular option due to their enhanced electrical, mechanical, and thermal characteristics. Although some of the polymeric materials have drawbacks in their mechanical and chemical characteristics, the decision to utilize a processing material is based on the suitability of its attributes for a particular application. For example, polyimide is often selected in situations that involve high temperatures and rough conditions due to its high heat and wear resistance, while polydimethylsiloxane (PDMS) is suitable in situations that benefit from its hydrophobicity and biocompatible nature. Therefore, although some of the issues faced by the polymeric materials include cracking and chemical inertness, the sensors made for healthcare and applications working at ambient temperatures perform better than their complementary rigid counterparts. Thus, the fabrication of flexible sensors has focused on their distinctive attributes and testing these in a wide range of applications. To develop the substrates, researchers have used different kinds of polymers depending on their Young’s modulus, ability to form composites, interfacial bonding with the nano-fillers, biocompatibility, hydrophobic nature, and resistance towards changes in temperature. Some of the common substrates employed for forming flexible sensors are PDMS [12,13,14], polyethylene terephthalate (PET) [15,16,17] and polyimide (PI) [18,19,20]. Similar to the substrates, the characteristics of the conductive materials must be taken into account when fabricating the sensors’ electrodes. Some of the common materials used to develop the electrodes for flexible sensors are poly(3,4-ethylenedioxythiophene) polystyrene sulfonate (PEDOT: PSS) [21,22,23], carbon nanotubes (CNTs) [24,25,26], silver [27,28,29], gold [30,31,32], copper [33,34,35], aluminium [36,37,38], and graphene [39,40,41]. These conductive materials have been used in different forms, such as nanoparticles, nanowires, nano-powder, and quantum dots. The conjugation of these polymers and conductive materials are processed with different kinds of fabrication techniques [42,43,44,45,46,47] to form the sensors. Some of the attributes that decide a sensor’s fabrication technique include its intended dimensions, repeatability, flexibility, stretchability, efficiency in terms of sensitivity, and whether it is intended to operate in harsh environments. Among these techniques, printing technology [9,48] has been frequently utilized by the researchers in recent times, as printed flexible prototypes have enhanced characteristics to a large extent. With printing technology, ultra-thin, light, and highly conductive sensors can be quickly fabricated on a large scale. Among printing technology, laser cutting is one of the popular ways in which substrates and electrodes are processed using a laser. Different kinds of lasers have been utilized to process the sensing prototypes depending on their distinctive parameters. Earlier in its development, laser cutting was performed only to glass, metals, and ceramics, but it has been optimized in recent times to work on polymeric substrates and nanoparticle-based electrodes. The number of parameters has also been increased to precisely optimize the features of the obtained sensing prototypes. The laser-cutting technique has been proven to be one of the better techniques among the available printing technologies [9,49] due to the reduced human intervention in sample preparation. Within the category of non-contact printing, laser cutting holds a higher value due to the avoidance of the requirement of any mask or template to form the design of the electrodes. This also avoids the effects of surface tension and the shadow effects of the printed inks on the flexible substrates. Due to these advantages with laser cutting, researchers are now trying to develop laser-printed flexible circuit boards, where—along with the sensors—the entire signal conditioning circuit is formed via laser printing [50]. Although many reviews have been conducted on printed flexible sensors based on their operating (electrochemical [51], strain [52], and electrical [53]) types, no previous article has presented a review based on the sub-categorization of the fabrication techniques in laser-assisted printing technology. In this paper, three important fabrication techniques in printing technology are presented: Nanocomposite-based, laser-ablated, and 3D-printed techniques. 

Although some earlier review papers [9,11] have examined work on printed flexible sensors, none of these have emphasized on one of those printing techniques. The authors give a review on the available printing techniques, but a substantial review of the fabrication of different kinds of sensors using one of the printing techniques is yet to be done. So, while some previous scholarship has focused on a particular material, this has limited focus on the versatility found in the available printing techniques [54,55,56]. Among the different kinds of printing techniques, laser-ablation technique has been chosen in this paper to highlight the different kinds of sensors that can be fabricated using it. Laser-cutting technique is one of the older and more popularly used techniques to process materials to develop sensors for a number of reasons. Firstly, this technique provides a quick and easy process to develop flexible prototypes. Secondly, a variety of sensors with varied electrical, mechanical, and thermal characteristics can be created using this technique. Also, this process can be utilized on different types of samples, as is outlined further below. This paper provides a substantial review of different categories of laser-cutting processing that can be exploited. A further classification has been done based on the type of samples where nanocomposite-based, laser-ablation, and 3D-printing techniques were highlighted. In each of these techniques, the agglomeration of different kinds of materials used to form the substrates and electrodes of the sensors has been explicated. A comparison highlighting the performances of the sensors fabricated with each of the subcategories is shown in Table 1. The limitations related to the raw materials and each of the sub-categorized fabrication techniques have also been explained in the final sections, along with some of the corresponding remedies to these bottlenecks.

The manuscript has been divided into four sections. Following the brief introduction given in Section 1, Section 2 elucidates the three categories of printed flexible sensors and some of the major work done on each is highlighted. Section 3 explains the challenges and issues presented by the current sensors that need to be addressed, along with some of the future opportunities regarding remedies to the present glitches and in response to market surveys. The conclusion of the paper is given in the final section of the paper.

## 2. Materials and Methods

The fabrication and implementation of printed flexible sensors have been broadly classified into three categories: Nanocomposite-based, laser-ablated, and 3D-printed prototypes. These categories are different with regards to their fabrication techniques and processed materials. The applications of the sensors developed from these three classes depends on the dimensions of the final prototypes, as well as their electrical, mechanical, and thermal characteristics. Some of the significant research that has been conducted on these three classifications is explained below.

### 2.1. Nanocomposite-Based Printed Flexible Sensors

The first category of printed sensors involves the laser curving of the electrodes to form the prototypes. This category can be sub-divided into two sub-categories based on the materials being processed under laser-ablation. The first class involves the flexible materials including polymers and nanoparticles, whereas the second class is related to the metal and metal oxides.

#### 2.1.1. Carbon Elements-Filled Nanocomposite-Based Printed Sensors 

**Figure 1:**
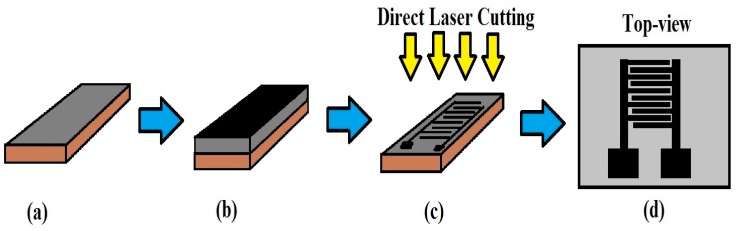
Fabrication steps of the CNT-PDMS-based sensor. (**a**) Casting and curing of polydimethylsiloxane (PDMS) on the poly (methyl methacrylate) (PMMA) template, (**b**) casting and curing a layer of the nanocomposite on the cured PDMS, (**c**) laser-ablation of the nanocomposite layer to form the electrodes of the sensor patch (**d**). Reproduced from Nag, A., Mukhopadhyay, S. C. and Kosel, J., 2016. Flexible carbon nanotube nanocomposite sensor for multiple physiological parameter monitoring. Sensors and Actuators A: Physical, 251, pp.148–155 [1].

One significant area of work as explained by Nag et al. [1] involves the use of polydimethylsiloxane (PDMS) and carboxylic acid (COOH) functionalized multi-walled carbon nanotubes (MWCNTs). The corrosion-resistant nature of the electrodes [65] and their better mechanical properties [66] are some of the advantages of this type of sensor patch. The functionalization of MWCNTs helps to increase the dispersion capability of the MWCNTs in the mixed polymer by increasing their interfacial bonding with the polymer. Figure 1 shows the steps followed to fabricate the sensor patches. The PDMS was cast on a poly (methyl methacrylate) (PMMA) template at a ratio of 10:1 between the base polymer and curing agent. PMMA as a template material offers a non-reactive nature and proper adherence with the cured PDMS. The height of the PDMS was adjusted to around 1000 microns with a casting knife. The PDMS was then desiccated for 2 h to remove any trapped air bubbles, then the sample was cured at 80 °C for 8 h. This formed the substrate for the sensor patch. A nanocomposite was formed by mixing MWCNTs with PDMS at a concentration of 4 wt.% of MWCNTs, which was found to provide a good balance between flexibility and conductivity. The conductivity of the patches was around 250 S/m, whereas their bending diameter was 14.8 mm. On the top of the previously cured PDMS, this nanocomposite layer was cast. The height was again adjusted by the casting knife to around 600 microns. The nanocomposite layer was desiccated and cured to solidify the substrate and then the electrodes of the sensor patch were formed by laser patterning the top layer with Universal Laser Systems. Three of the laser parameters–power, speed and z-axis–were optimized during each of the sensor fabrication processes. Power refers to the power of the laser beam; speed determines the laser nozzle’s rate of movement along the x and y directions, which defines the sensor structures; the z-axis is used to adjust the focal point of the laser on the sample. After the laser parameters were optimized, finally the values of 24 W, 70 m/min, and 1 mm, respectively, were used. The final sensor patch consisted of interdigitated electrodes with two pairs of electrode fingers, having a width and interdigital distance of 200 microns and 100 microns, respectively. Due to the flexibility of the patches, they were used for monitoring physiological parameters and respiration. Among physiological parameters, the sensors displayed a change in capacitance when they were attached to the elbow and knee of the limbs. The sensors were also attached to the lower part of the diaphragm to determine their contraction and expansion during respiration.

Another work, as explained by Massaro et al. [67], was related to the development of flexible tactile sensors via laser irradiation of plasmonic resonators. Along with the advantages of optical resonators, the key features of these sensors lie in their conjugated use for tactile sensing purposes. Due to the advantages of using gold and PDMS as conductive metal and polymers to form nanocomposites, the sensor provided an extra advantage of high flexibility and electrical conductivity. The nanocomposites were formed using PDMS and gold nanoparticles, which were subsequently laser-cut to form the optical layout for tactile sensing. Initially, micro-patterns that were composed using gold nanoparticles were shaped into PDMS-gold substrate using UV irradiation after placing a mask over it. After its deposition on the gold precursor solution, the mask was positioned on the precursor of the nanocomposite bulk surface. This allowed the formation of circular areas via laser-irradiation that contained gold plasmonic resonators. The minimum thickness of the plasmonic layer formed by the gold nanoparticles achieved was around 40 nm. This layer consisted of around 2–5 nanoparticle layers, each of which had a mean particle diameter ranging between 8 and 20 nm. The sensors were able to sense pressure down to around 0.5 kPa, which can be considered ideal for soft-touch sensing. 

**Figure 2:**
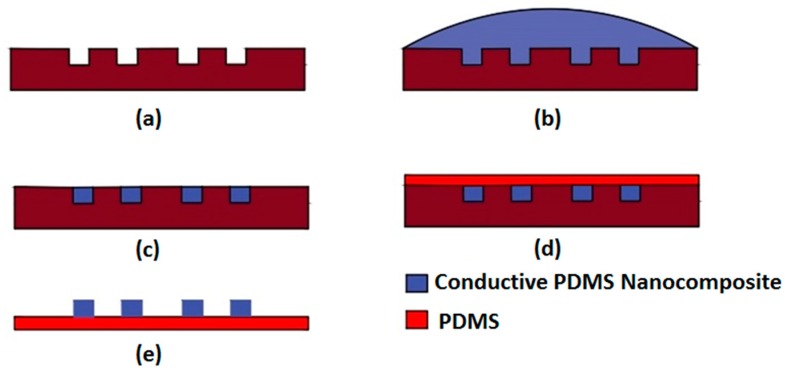
Schematic diagram of the hybridized fabrication process consisting of MWCNTs-PDMS-based nanocomposites. Reproduced from Khosla, A., Hilbich, D., Drewbrook, C., Chung, D. and Gray, B. L., 2011, February. Large scale micropatterning of multi-walled carbon nanotube/polydimethylsiloxane nanocomposite polymer on highly flexible 12 × 24 inch substrates. In Micromachining and Microfabrication Process Technology XVI (Vol. 7926, p. 79260L). International Society for Optics and Photonics [68].

Significant research has been conducted on the micro-patterning of flexible materials to form sensing purposes. One of them has been explained by Khosla et al. in regards to the fabrication of arrayed-sensors on flexible 12 × 24 inch substrates from MWCNTs-PDMS nanocomposites [68]. The difference of this methodology from that of the first one explained in this section lies in the use of micro-contact patterning which helps in differentiating the intensity of the induced-strain corresponding to different locations. Another difference is the resistive nature of these sensors, which makes them have quicker responses and a higher range of resistance. High-frequency ultra-sonication was used to prepare nanocomposites, which were then patterned on a PMMA template. Laser-ablation was used to process the PMMA template to form sheets of defined sizes. This technique has proven to be useful to form nanocomposites of low percolation threshold, forming a large array of microelectrodes. The process was initiated with the suspension of MWCNTs on heptane solution and then subsequently mixing it with PDMS via ultra-sonication. The micro-folds were formed using a CO_2_-based laser, which had a rated power of 60 watts and speed of 1143 mm/s. After the nanocomposite was cast on the micro-folds, the excess amount was scrapped off. A layer of PDMS was added on top of the cast nanocomposite, which was then cured and peeled off to form the sensors. The characterization of the sensors was done based on the change in the resistivity with respect to the changes in the weight percentages of MWCNTs in PDMS matrix. This work can be compared to [1], where similar work based on the formation of nanocomposites using an optimized value of 4 wt.% of MWCNTs in the MWCNTs/PDMS matrix. The measurement of the individual electrodes in the array with similar dimensions were also done to show that the resistances of these electrodes were around 9.93 Ωm. Figure 2 shows the schematic diagram of the individual steps of the fabrication process [68].

Rouhollah et al. [69] explained the fabrication of strain sensors via direct laser writing on freestanding nanocomposites. The design of the sensors was made as 3D, freestanding, patterned strain-sensors formed with single-walled carbon nanotubes (SWCNTs). The key features of this fabrication would be the consideration of free-standing nanotubes that were advantages in increased volume, increased energy density per unit area, and mechanical flexibility. Also, due to the free-standing nature of the SWNCTs, these sensors had an extra advantage of reacting to strains of much smaller magnitude in comparison to other CNTs-based strains sensors. The laser cutting was done using ultraviolet-assisted direct-writing process. The SWCNTs being synthesized with UV laser ablation were mixed with UV/heat-curable epoxy that contained a UV photo-initiator. The final steps involved the curing of the composites at 30 °C and 50 °C for 12 h and 14 h, respectively. The first type of strain-sensor consisted of four identical micro-springs having a diameter of 1 mm. A small circular aluminum microplate with an inter-coil diameter of 2 mm was used as the substrate and top layer for the micro-springs. Both the sensors were cured at 120 °C for an hour to complete the fabrication process. The sensors were subjected to strain of different magnitudes to determine the change in current as a function of the applied voltage. The sensors were capable of responding to small mechanical loading, which made them advantageous for applications like tactile-sensing. The 3D-printed nature of these micro-structured sensors provided a high sensitivity due to the presence of the four parallel micro-springs, which gave them an extra advantage in comparison to the some of the conventional methods used to develop strain sensors like photolithography [70] and screen printing [71] techniques.

**Figure 3:**
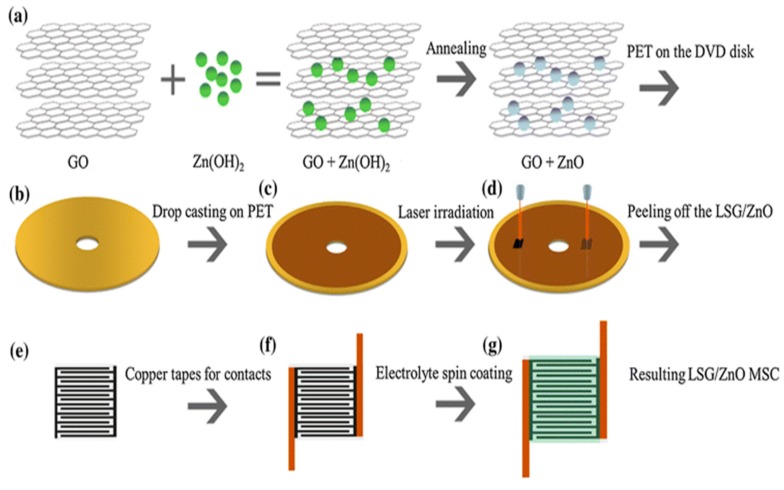
(**a**) Schematic representation of the fabrication of ZnO/GO nanocomposites. (**b**–**g**) Steps depicting the formatting of micro-supercapacitors. Reproduced from Amiri, M.H., Namdar, N., Mashayekhi, A., Ghasemi, F., Sanaee, Z. and Mohajerzadeh, S., 2016. Flexible micro-supercapacitors based on laser-scribed graphene/ZnO nanocomposite. Journal of Nanoparticle Research, 18(8), p.237 [72].

The fabrication and characterization of micro-supercapacitors was explained by Amiri et al. [72], where laser inscription was used on nanocomposites to form the electrodes. In accordance with a lot of work done on laser-scribed graphene, the significance of this work lies in the use of the fabrication of micro-supercapacitors. In the addition of zinc oxide, reduction of the resultant noise leads to higher operating temperature due to their enhanced electrical properties and high surface-to-volume ratio. Another advantage of this sensor is its enhanced electrochemical properties in comparison to other [73,74] nanocomposite-based supercapacitors. The electrodes were developed from graphene and zinc oxide nanocomposites. Reduced-graphene oxide (rGO) was mixed with zinc oxide (ZnO) nanoparticles on a DVD to form a homogenous solution. GO was developed using a modified Hummer’s method, which was then added to NaNO_3_ and H_2_SO_4_ solutions. Different mass ratios between the ZnO and GO solutions were considered to form the optimized nanocomposites. The diameter of ZnO nanoparticles was kept constant between 20 and 50 nm. The solution was heated in a vacuum furnace and subsequently spin-coated, forming a homogeneous solution. The suspension was then drop-casted on a laser-scribing DVD, with a wavelength and spot size of 780 nm and 20 µm, respectively. The resultant composite was glued on a PET substrate where the electrodes were formed using copper wires connected with silver paste. Figure 3a–c depict the fabrication process of the ZnO/rGO nanocomposites [72]. The performances of the sensors were evaluated for electrochemical sensing with a three-electrode system using cyclic voltammetry where different concentrations of zinc nitrate were considered to generate different mass ratios of the resultant nanocomposites between ZnO and rGO. The three-electrode system had a platinum rod and standard Ag/AgCl electrodes as a counter and reference electrode. 

The use of laser ablation on nanocomposites is also explained by Liu et al. [75] in their work on conductive composites developed from CNTs and PDMS. The key advantages in these sensors lie in the high electrical conductivity of the electrodes, flexibility of the sensors, and ability to achieve features in micron levels. In comparison to other sensors explained in this paper that involve CNTs and PDMS, the specialty of these sensors involved the increase in the versatility in the piezoresistive sensors to achieve a minimal feature below 20 microns. Laser-cutting was done on PET molds to fill them with an appropriate amount of CNTs-PDMS using smooth-edge tooling. The advantages of using PET as molds for casting the processed materials lies in their low cost, impassive nature towards PDMS, ability to withstand high temperature, and high reproducibility. PET polymer was laser-cut with a homogenous laser beam to create specific patterns. An imaging mask was used on top of the PET surface to define the size and shape of the drilled holes. The dimensions of the holes were de-magnified by an optical lens that was placed between the imaging mask and PET surface. After attaching the PET surface on a glass surface, the substrate was treated with chlorotrimethylsilane to assist proper de-bonding of PDMS from PET substrate. The casting was done on the laser-patterned PET surface using nanocomposites developed with MWCNTs mixed with PDMS. The sample was then cured in the presence of de-bonding device on top of the bulk PDMS, followed by taking off the de-bonding equipment from the PET surface. The high compatibility of the PDMS aided the firm attachment of the nanocomposite to the de-bonding equipment. The thickness of the nanocomposites was made identical to the depth of the molds, which was verified using a reliable replication process. 

Wu et al. [76] showcased the self-healing capability of nanocomposites using infrared laser. These sensors are important due to their structural restoring nature and mechanical robustness. The nanocomposites were developed with functionalized graphene nano-sheets (FGNS) and polyurethane-based on Diels Alder chemistry (PU-DA). The tune of the mechanical breaking strength of the nanocomposites was varied by altering the amount of FGNS. The composites had a volume resistivity of 5.6 × 10^11^ Ωcm with a maximum loading of 1 wt.%. The presence of FGNS in the nanocomposites increased their capability for IR absorption in comparison to the PU-DA matrix. The change in loading from 0.5 wt.% of the FGNS created a change in Young’s modulus and tensile strength to 127 MPa and 36 MPa, respectively with a break elongation of 1100%. The increase in the filler content also affected the temperature, increasing it rapidly and to a higher end. The electrical conductivity of the device could be recovered by drop-casting silver paste on the composite substrate and using IR laser irradiation on it. The irradiation time and wavelength used by the laser were 1 min and 980 nm, respectively. The intention behind these composites was to mimic human skin to depict its self-healing property. GO was synthesized by using Hummer’s method, followed by mixing it with hydrochloric acid to form ethanol-amine-functionalized graphene. Poly (tetramethylene glycol) (PTMG) was added to DMF at a ratio of 4:15 and attached to 4,4-diphenylmethane diisocyanate (MDI)/dimethylflouride with magnetic stirring under an elevated temperature and nitrogen atmosphere. The mixture was then pressed with Teflon plates under 60 °C in a convection oven and 100 °C in a vacuum oven for 12 h and 1 day, respectively. A healing capability of 96% was achieved for these sensors, which is significantly similar to human skin. 

**Figure 4:**
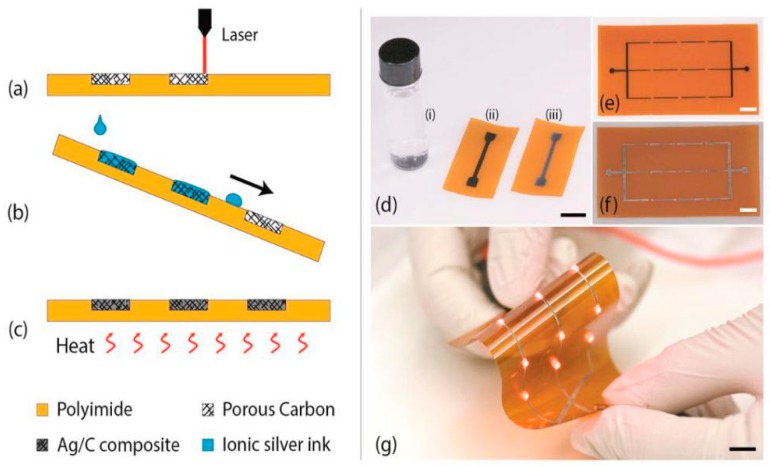
(**a**–**c**) Schematic diagram of the fabrication steps using laser pyrolysis, (**d**) laser-carbonized traces prior to and after the coating with silver nanoparticles, (**g**) experimentation with an array of LEDs. Reproduced from Rahimi, R., Ochoa, M. and Ziaie, B., 2016. Direct laser writing of porous-carbon/silver nanocomposite for flexible electronics. *ACS applied materials & interfaces*, *8*(26), pp. 16907–16913 [27].

Rahimi et al. [27] discussed the direct laser writing of carbon/silver nanocomposites on flexible substrates. The highlights of this work is related to the conjugation of the porous carbon nanomaterials and silver nanoparticles to form the electrodes of the sensors. CO_2_ laser-cutting was done on a polyimide sheet to generate carbonized compounds for adding selective laser silver deposition on them. Another highlight of this paper was the very low sheet resistance of the composites, ranging between 50 and 0.02 Ω/cm with less than 0.6 Ω variation in resistance, even after more than 150,000 bending cycles. The bending radius of curvature was 5 mm with prominent changes in the sheet resistance with respect to the change in temperature from 27 ˚C to 120 ˚C. The sensors were employed for pressure sensing ranges between 0 to 97 kPa with a sensitivity of −26 kHz/kPa. Laser pyrolysis of a polymeric substrate was done to create a porous network on the flexible substrate containing conductive carbon traces. The traces were made selective using reactive silver ink. The optimized laser parameters values operating with a beam spot size of 50 µm on continuous wave mode were 100 W, 2 m/s, 10 microns for power, speed, and wavelength, respectively. The highest conductivity of the porous-carbon was achieved with the laser being operated at a power of 6.75 W and scanning speed of 1.3 m/s. The selective coating of the carbon was done to enhance the latter’s electrical conductivity and robustness. The silver solution was prepared by mixing silver acetate and ammonium hydroxide at defined ratios, followed by ultra-sonicating it for 30 min. Figure 4a–g shows the schematic diagram of the fabrication process, the silver layer coating and their experimentation with simple circuits of LEDs. The resulting nanocomposites and LEDs were able to withstand mechanical bending while preserving the same intensity. The sensors also operated as wireless flexible sensors for wearable and implantable applications by operating as an LC passive pressure sensor. 

Arorami et al. [77] discussed the fabrication of compliant electrodes using nanocomposites developed from PDMS and carbon compounds. The electrodes of the sensors were robust and stretchable in nature, being developed from laser-ablation technique. One of the highlights of this work can be related to the high resolution of the of the electrodes that were developed in large areas, forming an array of sensors. Casting carbon-PDMS composite layers with thicknesses ranging between 2–50 µm was done, followed by laser-ablating them to form the electrodes. Interdigitated electrodes were formed operating as capacitive touch sensors.

**Figure 5:**
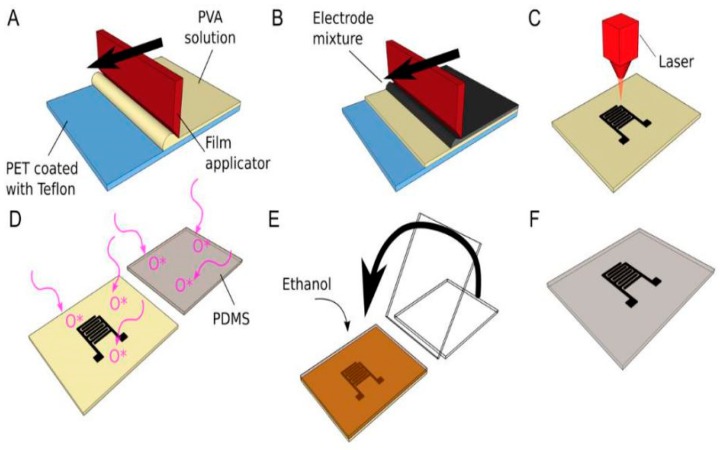
(**a**–**f**) Schematic representation of the fabrication process of the carbon-PDMS composites. Poly (vinyl alcohol) (PVA) aqueous solution followed by carbon/PDMS composites were cast on the PET substrate and cured to solidify the conductive material. Laser-cutting was done to the composites and they were attached to the oxygen plasma-treated PDMS to form the sensors. Reproduced from Araromi, O.A., Rosset, S. and Shea, H.R., 2015. High-resolution, large-area fabrication of compliant electrodes via laser ablation for robust, stretchable dielectric elastomer actuators and sensors. ACS applied materials & interfaces, 7(32), pp.18046–18053 [77].

These electrode-shaped composites were then covalently bonded with a PDMS layer using an oxygen plasma activation technique. Poly (vinyl alcohol) (PVA) aqueous solution was cast on a PET substrate with a length and width of 29 cm and 20 cm, respectively. The composite electrodes made from carbon black and PDMS were cast on the PVA substrate and then the sample was subsequently cured in the oven. The cured composite was then peeled off the PET substrate and laser-cut to form the electrodes. These electrodes were then placed in contact with PDMS that was treated with oxygen plasma in a pressure chamber with power, frequency, and pressure of 20 W, 13.56 MHz, and 0.2–0.3 mbar, respectively. The sensors responded to a stretching of over 25% strain, displaying high sensitivity. These sensors were employed both for capacitive proximity sensing and as an elastomeric membrane working on the change in its dielectric behavior due to the change in its permittivity. The change in capacitance of the sensors was measured with the change in their radius of curvature when they were attached on different objects, like a coffee mug. The fabrication process of the carbon-PDMS composite-based sensors is shown in Figure 5 [77].

Further research has been conducted on nanocomposite-based printed sensors involving the development of recyclable and malleable sensors that resemble electronic skins with regards to their functionalities and mechanical properties [78]. Researchers have been working on electronic skins, or e-skins [79,80], to develop sensors that would exactly mimic human skins in terms of their flexibility, stretchability, and self-healing capabilities. One of the major significant advantages of these sensors is the re-healing capability in room temperature, which has not been seen in any of the developed electronic skins so far. The conjugation of these flexible e-skins with some controlling systems like artificial intelligence is something that researchers need to work on. The sensors were developed via the doping of thermoset polyimine with silver nanoparticles. The conductive polyimine films were formed by mixing terephthalaldehyde, diethylenetriamine, tris (2-aminoethyl) amine, and silver nanoparticles with ethanol. The mixture was sonicated in the presence of PDMS, evaporated, and heat-pressed. The conductive films were then subsequently integrated onto polyimine substrate to form covalent bonds between the sensors and the substrate. Laser-cutting was done on the polyimine films, which operated on four-probe measurements to determine the change in resistance due to temperature, flow, and humidity. Serpentine design of the electrodes was considered on the sensors to minimize the effects of the induced-strain. While the sensors were broken due to mechanical cutting when mounted on the human arm, they were re-healed with the help of a re-healing agent. The re-healing agent was applied on the cracks and subsequently heat and pressure was applied on it at 80 ˚C for 8.5 kPa respectively. The sensors regained their complete functionality in terms of their sensing capability and mechanical integrity and the re-healing agent was applied to it and subsequently pressed. The recyclability of the sensors was achieved by soaking them in a solution made of oligomers and monomers that degraded the polymer matrix and mixed them with ethanol. The recycled solution and nanoparticles were then used again to create new electronic skins. The developed e-skins were conformed onto the different surfaces with a curved nature. The change in resistance and capacitance values were measured with respect to weight, bending cycle, temperature, and humidity. 

#### 2.1.2. Metal-Filled Nanocomposite-Based Printed Sensors

**Figure 6:**
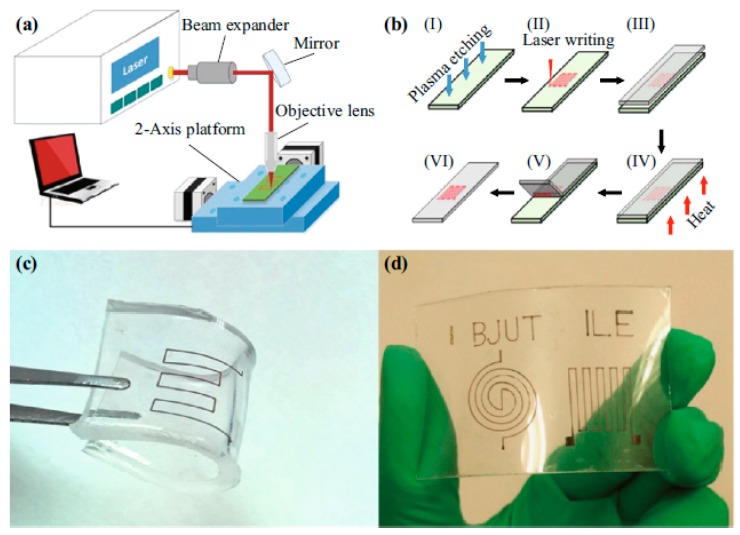
Schematic diagram of the (**a**) experimental setup and (**b**) steps of fabrication for developing the sensors. The steps of fabrication included plasma etching, laser writing, coating, curing, peeling, and overlay of the specified processing materials to develop the sensors. Images of the sensors consisting of (**c**) copper electrode and PDMS substrate and (**d**) Spider and zigzag electrode designs on the flexible substrates [81].

Bai et al. [81] fabricated flexible strain sensors of copper electrodes by using laser direct writing method for health monitoring. Copper oxide nanoparticles were printed on stretchable polydimethylsiloxane (PDMS) substrate and finally the electrode integrated PDMS based strain sensor was cut into a size of 5 × 2.5 cm^2^. This sensor exhibited excellent performance in sensing human motion with ultrahigh sensitivity, under both compressive and tensile strains. This research group has achieved an electrical resistivity of 96 μΩ at a certain thickness. Figure 6 shows the schematic diagram of the experimental setup and steps of fabrication followed to form the copper oxide/PDMS-based sensors [81]. 

Kravchuk et al. [82] printed a strain sensor of silver nanoparticle on flexible polyimide substrate silver structures by laser-sintering with a continuous wave of 405 nm. Due to its small heat-affected zone and efficient energy deposition, the laser-sintering process was used over the ink-jet printing process. Subsequently deeper-lying particles could also be sintered and diffused by the radiation of the beam, with a power of 200 mW and a scanning speed of 14 mm/s. The gauge factor of the thermal-sintered was 2.9, whereas the laser-sintered strain gauge of lased-sintered strain-sensor gauge was around 3.6; the laser-sintered gauge showed a stable output value. Reduction of the thermal loading due to short processing times was another advantage of this sensor.

Mizoshiri et al. [83] fabricated temperature sensors using femtosecond fiber laser-reduction patterning of copper oxide nanoparticles and copper-rich micro-pattern at laser scan speeds of 1 mm/s and 15 mm/s and pulse energies of 0.54 nJ and 0.45 nJ, respectively. The electrical resistivity of 8-μm-film thickness of both the materials have 10 Ωm and 9 μΩm as output parameters, respectively. Nakajima et al. [84] prepared a spinel Mn–Co–Ni oxide film thermistor by dispersion of nanoparticles using pulsed laser-induced liquid-phase sintering process in air. The photoreaction of the laser radiation was carried out on a polyethylene terephthalate sheet as a substrate during the coating of a flexible sensor. Since the oxide film had a nature of crystallization by the laser radiation, it could be used as a temperature sensor. Also, the sensors could respond rapidly at a certain temperature of 4429 K. 

### 2.2. Laser-Ablated Printed Flexible Sensors 

The second category of the printed flexible sensors was developed by laser-ablation. The laser-ablation processes shown below were carried out mostly on the surface of the sensor itself to form and analyze the prototype. The significance of these types of sensors lies in their increased efficiencies and sensitivities due to their capability to work on a wide range of processing materials. The use of laser-ablation is an alternative to the conventional photolithographic technique to form scalable and straightforward sensors.

**Figure 7:**
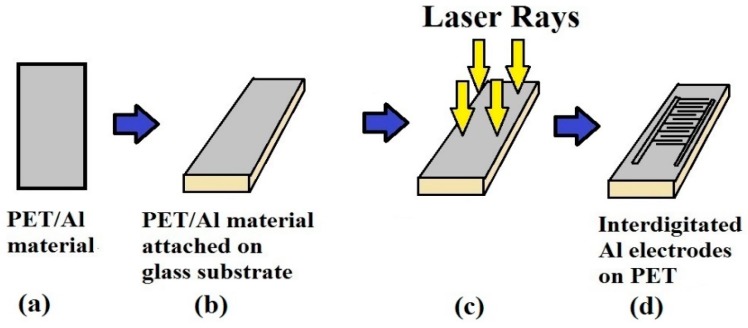
Schematic diagram for the fabrication of the Al–PET sensors. (**a**, **b**), The metalized polymer film was attached to the glass substrate, (**c**) direct laser cutting on the metallic side of the film formed the interdigitated electrodes, (**d**) on the sensor patch. Reproduced from Nag, A., Mukhopadhyay, S.C. and Kosel, J., 2016. Tactile sensing from laser-ablated metallized PET films. IEEE Sensors Journal, 17(1), pp. 7–13 [2].

The work explained by Nag et al. [2] was based on the laser-ablation of metalized polymer films to form the sensor patches. The conductive side of the metalized PET films was scanned to form the electrodes of the sensor patches. A single-step fabrication method and the high sensitivity of the electrodes towards forces [85] are the two main advantages of this sensor patch, which has high flexibility characterized by a bending diameter of 10 mm. A 500-microns-thick polymer was coated with aluminum 300 microns thick. The metalized PET film was attached to a glass substrate with biocompatible tapes, with the metallic side facing upwards. The metal film was then patterned into interdigitated electrodes with the laser. The width of the electrode lines was 40 µm with a distance of 150 µm between the consecutive fingers, which resulted in a resolution with reasonable quality. A schematic of the fabrication process is shown in Figure 7. The sensor patches were patterned with laser parameters of 12.6 W, 52.5 m/min, and 1.2 mm for power, speed, and z-axis, respectively. Twelve pairs of electrode fingers were developed, each with a length and width of 1.2 mm and 41 microns, respectively. The sensors were used for tactile-sensing where pressure was manually applied on the sensing area of the patches to determine change in their responses. The index finger, palm, and thumb were used to determine the responses for pressures ranging between 540 Pa to 540 kPa. 

The study by Rahimi et al. [86] involved the laser ablation of metalized paper (MPs) substrates to form inexpensive paper-based sensors. The use of metalized polymer films has been very popular nowadays due to the quick and reduced cost of fabrication, enhanced features in terms of higher robustness, and increased tolerance level towards heat in comparison to individual metallic films. This work is very different from all the research works presented in this manuscript as it highlights the study done on the different types of laser ablation done on flexible substrates with two types of lasers. The distinction between the lasers can be based on their specifications like power, speed, wavelength, mode of operation, and spectral bandwidth. Direct laser ablation (DLA) and indirect laser ablation (ILA) were tested on the MPs at wavelengths of 1.06 with an Nd: YAG and 10.6 microns with a CO_2_ laser, respectively. The CO_2_ and Nd: YAG lasers operated on the continuous wave and pulsed mode respectively. The difference between DLA and ILA lies in the removal of the paper substrate while etching the aluminum layer.

**Figure 8:**
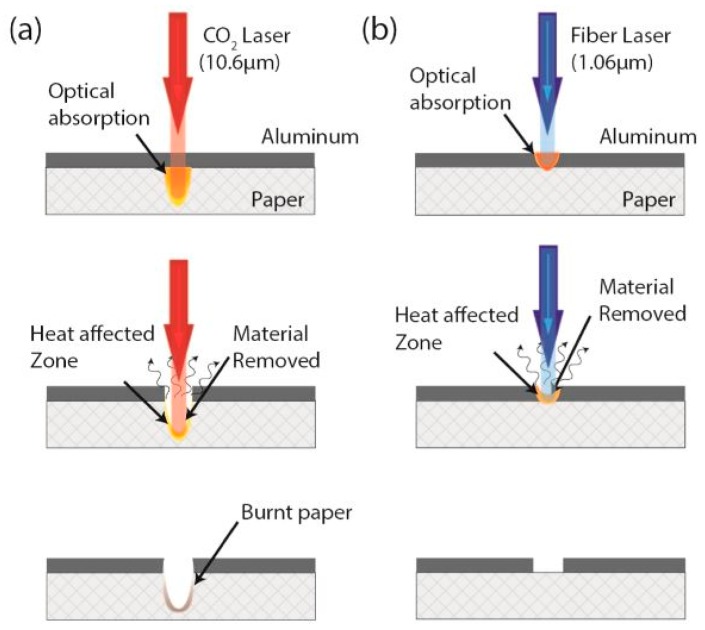
Illustration of the laser ablation of a thin aluminum layer on metalized paper (MP) using (**a**) indirect laser ablation (ILA) using CO_2_ laser and (**b**) direct laser ablation (DLA) using Nd: YAG laser. Reproduced from Rahimi, R., Ochoa, M. and Ziaie, B., 2018. Comparison of Direct and Indirect Laser Ablation of Metallized Paper for Inexpensive Paper-Based Sensors. ACS applied materials & interfaces, 10(42), pp. 36332–36341 [86].

ILA operates with a narrow set of laser settings to avoid causing any damage of the paper substrate. DLA operates with defined laser settings to prevent damaging the mechanical and nano-fibular structure of the paper substrate. Figure 8 shows an illustration of ILA and DLA lasers [86]. When the sensors were operated for humidity sensing in the range of 2%–85%, it was found that the sensors fabricated with DLA had a superior performance in comparison to ILA. The raw material used for the fabrication purposes was a cellulose film with an average thickness of 56 microns, having a vacuum-deposited aluminum coating of 25 microns. The difference in the results from the two types of laser beams was due to the strength of the material to absorbing each of the laser beams. In these experiments, the absorbance of aluminum for the Nd: YAG laser was higher than that of the CO_2_ lasers. This is because the maximum absorbance of the laser beams from the CO_2_ lasers took place on the cellulose-supporting substrate. The photon energy of the laser beam was one of the deciding factors for the absorbed portion of the energy. The mechanical strength of the materials was studied using a wet-etching process, whereas the cost-effectiveness process was studied using ink-jet printing and screen-printing processes. Interdigitated electrodes were developed on each of these types, having three different kinds of configurations These two types of sensors were then used for determining the change in their capacitance values with respect to the change in relative humidity. The humidity values were varied between 2% and 40% to show their responses for the different types of configurations for each of the type of sensors. 

**Figure 9:**
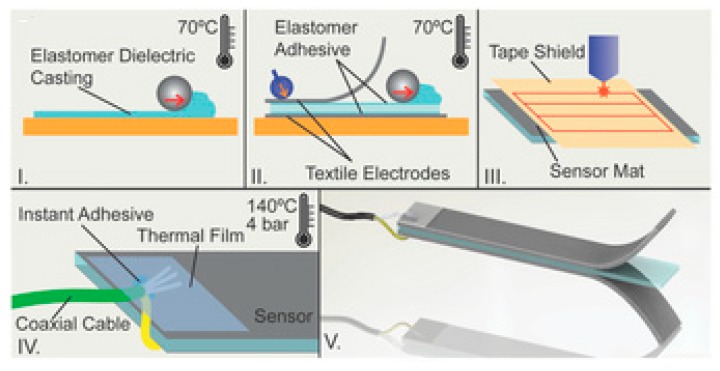
Schematic diagram of the manufacturing procedure of the composite textile-silicone sensor: (**i**) Dielectric silicon casting, (**ii**) connecting fabric electrodes using silicone elastomer casting, (**iii**) attaching tape shield and laser cutting of sensor, (**iv**) formation of a permanent electrical connection between coaxial cable and fabric electrode using adhesive and thermal film, and (**v**) 3D-representation of the sensor. Reproduced from Atalay, A., Sanchez, V., Atalay, O., Vogt, D.M., Haufe, F., Wood, R.J. and Walsh, C.J., 2017. Batch fabrication of customizable silicone-textile composite capacitive strain sensors for human motion tracking. Advanced Materials Technologies, 2(9), p. 1700136 [91].

A low cost and highly self-contained sweat sensor was developed by Rose et al. [87]. They proposed an adhesive radio-frequency identification (RFID) sensor [88] patch that can be made compatible with human skin. This work has proved to be useful for amalgamating the work on laser-induced flexible sensors with wireless sensing technology, which not only helps in monitoring from distant locations, but also in its multi-functionality and at increased speeds. They are similar in the consideration of substrates of this work with [62], with two differences. One was the formation of the electrodes in that work directly on the substrates by 3D-printing and the second was the substrates in this case had a protective layering, which not only looked like bandages, but the idea behind developing this sensor can be assigned to the fabrication of a wireless hydration sensor. All of the layers of these sensors were formed by laser-cutting. The patches here were used as wearable-sensing prototypes and all unused areas of gas/liquid resistant polyimide substrate film were removed to offer skin access and to increase the breathability of the sensor bandage. Laser-cutting was done using a Universal Laser Systems VLS3.50 CO_2_ laser cutter. The sensing device involved skin adhesives, electronics, paper microfluidics, and a vapor-porous adhesive textile [87]. Potentiometric sensing was carried out to determine the in vitro tests done with specified concentrations of certain solutions containing like sodium, chloride, potassium, magnesium, ammonium, and zinc ions. The responses were calculated in terms of voltage with an accuracy of 96%. The sensors were connected for monitoring purposes to an Android smartphone app, where the responses toward the change in sweat and surface temperature were sent. This work can be compared with the work displayed by Atalay et al. [89,90], where the fabrication of a stretchable textile–silicon capacitive sensor was reported. The sensors were used for articulation detection in human, soft robotic applications, and exoskeletons [91]. The developed sensors were made with conductive fabric as an electrode and silicone elastomer as a dielectric. The batch fabrication technology allowed the creation of a large sensor mat and random shaping of sensors, which was completed accurately using laser-cutting of the sensor mat. The developed capacitive sensors showed good linearity and a gauge factor of 1.23. These sensors were integrated into a glove for monitoring finger motions. Figure 9 represents the schematic diagram of the fabrication process of the composite textile-silicon sensor [91].

A study by Acuautla et al. [92] regarding the use of direct laser-patterning is related to the fabrication of the gas sensor on a flexible substrate. These sensors also used zinc oxide nanoparticles, but as a commercial to determine the performances of the fabricated sensors developed from titanium-platinum interdigitated electrodes. Although the comparative results showed that the response of these sensors were close to that of the commercial ones, the disadvantage of the high cost of platinum and titanium makes these sensors cost-ineffective. However, the sensors showed highly repeatable responses when they were tested for the gas-sensing applications. The sensors used polyimide films 75 µm thick as substrates, which were initially treated with oxygen plasma in order to improve its surface quality in terms of the adhesiveness towards the sputtering metals. Followed by the sputtering of metals at defined thicknesses, a femtosecond-diode-pumped ytterbium amplified laser, with an operating wavelength and spectral bandwidth of 1030 nm and 5 nm, respectively, were used for performing the pulse-mode operation. The finite element was used to validate the platform via thermal simulation to obtain a homogenous temperature around the sensing area. A set of galvo mirrors and f-theta-lens was used to pass the laser beam with a focal length of 254 nm. Shock-wave assisted delamination process was then used to recover the removed metal layer from the substrate. The sensors were used for monitoring changes in ammonia and ozone gas in the presence and absence of commercial ink of ZnO nanoparticles at different concentrations and temperatures. The change in the resistances values was calculated with respect to time duration for concentrations of ozone and ammonia varying from 10 ppb to 300 ppb. 

Another study in relation to the laser-ablation was based on the laser-patterning on metalized films and using plastic foils as flexible substrates [93]. The significance of this work lies in the first precise measurement of the threshold ablations for platinum metal. Some of the advantages of Kapton films over other commonly used flexible substrates like PET include their high operating temperature and resistance toward changing temperatures. Laser-ablation of platinum and silver films 100 nm thick was carried out with Kapton films 50 microns thick as thin-film substrates. The specifications of the laser parameters in terms of pulse duration, frequency, and wavelength were 500 fs, 1000/10,000 Hz, and 1030 nm, respectively. The deposition of the silver layer was carried out using spin-coating and annealing methods. The metal layers were deposited on the Kapton layer via radio-frequency (RF) sputtering process without the need of any thermal post-process. The conductivity of silver obtained using laser-ablation was higher than that of ink-jet printing. The optical properties of the laser-ablated films, especially for platinum, was the exception, as it showed a reflection of 92.6%. The reflection intensity of platinum on Kapton tapes was also the same as that of the bulk material. 

Dankoco et al. [94] outlined the fabrication of laser-ablated sensors for developing prototypes for temperature sensing. The prototypes were used as resistive temperature devices (RTDs) to measure the temperature of the human body. Kapton tapes were used as substrates where platinum, silver, and copper were sputtered via RF magnetron sputtering, with thicknesses of 100 nm, 200 nm, and 200 nm, respectively. The adherence of the sputtered metal to the substrate was improved by treating the Kapton tapes with oxygen plasma before the sputtering process. A femtosecond laser was used for laser-ablation, operating on a frequency and spectral bandwidth of 1030 nm and 5 nm, respectively. Similarly, Bellini et al. [95] proposed the use of laser ablation for the customized production of flexible electronic devices. Along with the progress in the fabrication of flexible sensing systems, the possibility for the development of thin-film transistors is always advantageous due to the complexity in their structure along with their functional advantages [96]. Another advantage of this work can be regarded as the formation of the shortest channel length in the thin-film transistors, which would offer enhanced performance in terms of sensitivity and reproducibility. Here, laser-processing was used to fabricate the flexible display organic thin-film transistor (OTFT) backplanes. PET/PEN polymers were used as substrates where gold-coated organic planarization layers were formed to obtain dielectric layers. Ultrafast diode-pumped solid-state (DPSS) lasers were used for the laser-ablation process, having two separate setups for fabricating the sensors. The reason for considering two setups was to determine the comparatively optimized settings for forming the source-drain channel. The two wavelengths for the setups were 1026 nm and 532 nm, having a pulse duration of 220 fs. The researchers figured out that laser-ablation was circumvented when the power and z-axis were varied from −225 µm to −325 µm and from 9.5 gJ to 15.5 gJ, respectively. The source and drain electrodes of the organic transistor channels were developed along with the etching of the thin metal layer, which was 80 nm thick, to form a channel 5 microns wide. It was seen that highly irregular channel edges were created when a pulse was injected over a surface, with pulses ranging between 25–250 pulses per area. A 100% yield was obtained on the metal layer along with proper electrical connections on the backplane transistor stacks.

Acuautla et al. [97] outlined the work done on the fabrication of flexible ozone sensors via the conjugation of photolithographic and laser-ablation techniques. The integration of two different conventional fabrication techniques and the development of sensors for ozone detection can be highlighted as the significant parts from the perspective of fabrication and application. The laser-ablation was done on polyimide substrates, in the form of Kapton tapes. The choice of Kapton tapes was made due to their flexible nature and high resilience toward changes in temperature. Interdigitated electrodes were developed on the sensors with a sensing area of 4000 × 2500 microns. The electrodes were made up of titanium and platinum with thicknesses of 5 nm and 100 nm, respectively. The micro-sensing area was opted to have an interdigital distance of 60 microns to form an overall surface area of 2200 × 900 microns. After the oxygen plasma treatment of the substrates, followed by sputtering of titanium and platinum, laser-ablation was carried out with a femtosecond-diode-pumped ytterbium amplified laser, with an operating wavelength, spectral bandwidth, and pulse duration of 1030 nm, 5 nm, and 350 ± 20 fs respectively. The final electrodes developed with this process had a thickness and length of 100 nm and 90 microns, respectively, and a cross-section of 1.98 µm^2^. The heater in the sensor had a length of 5.36 mm. After the development of the flexible sensors, ZnO nanoparticles were drop-casted on the sensing area of the prototypes to make them sensitive toward ozone gas. Followed by cleaning the sensors with acetone and ethanol, drop-casting of ZnO nanoparticles with a thickness of 280 nm was done on them at room temperature. Finally, the sensors were annealed at 300 °C for 3 h to enhance the film density, quality, and stability of the sensitive material. The sensing of ozone gas was done by determining the change in resistance in the presence of the gas. The concentrations the gas was varied between 5 ppb and 300 ppm, depicting a fast and reversible response. They showed an optimize resistance at an elevated temperature of 200 ˚C. The response and recover times for the sensors were 4.5 min and more than one minute, respectively. 

**Figure 10:**
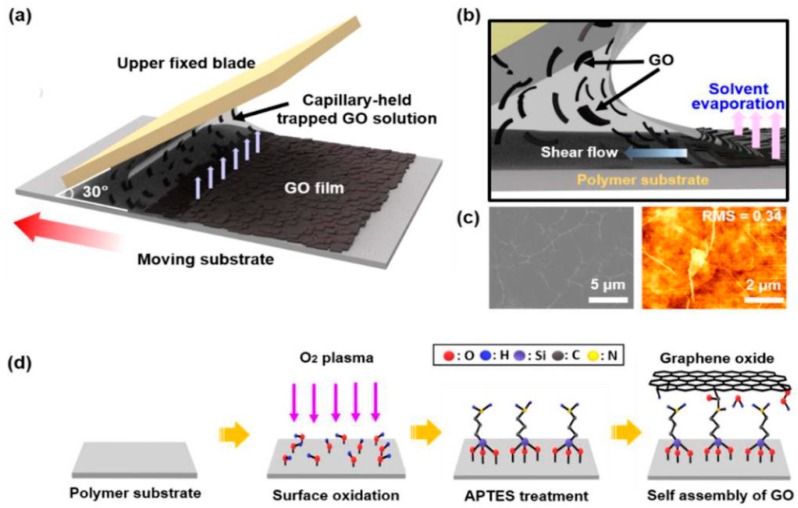
Steps of fabrication of the laser-ablated reduced-graphene oxide-based thin film via flow-enabled self-assembly. Reproduced from Park, R., Kim, H., Lone, S., Jeon, S., Kwon, Y., Shin, B. and Hong, S., 2018. One-Step Laser Patterned Highly Uniform Reduced Graphene Oxide Thin Films for Circuit-Enabled Tattoo and Flexible Humidity Sensor Application. Sensors, 18(6), p.1857 [98].

One of the interesting works regarding the one-step laser-ablation process was explained by Park et al. [98], where reduced-graphene oxide thin films were processed to form flexible sensors. The highlights in these sensors lie in the tattooed nature of the sensors, which not only increased the effective contact area, but also caused a reduction in the impedance between the sensor and the tested material. Graphene oxide and polypropylene were used to develop electrodes via depositing them on polypropylene (PP) substrate. Followed by the fabrication of GO using Hummer’s method, GO was mixed in deionized water to form a uniform dispersive solution. To ensure proper adherence between the PP and GO solution, self-assembled monolayer (SAM) was developed on the hydrophobic polymeric substrate via initial washing of the PP with acetone, DI ethanol, and isopropyl alcohol to reduce the amount of contaminants present on the surface. Then, PP substrate was exposed to oxygen plasma and subsequently treated with a 3-aminopropyltriethoxysilane solution for one hour at an ambient temperature. This finally formed the terminal amine groups on the PP surface. The injection of GO solution on the confined space was done to form a capillary-based meniscus. The final step included the coating of PP film with the translation of the colloidal solution at a constant speed of 15 mm/s for around 100 cycles. The laser induction was done on the rGO films to form the electrode designs. The laser parameters were set to a frequency, pulse duration, and power range of 30 kHz, 20 ns, and 0.04–0.8 W, respectively. Figure 10a–d shows the surface modifier of the PP substrate and formation of the laser-ablated reduced-graphene oxide-based thin films. The sensors were then opted for humidity-sensing applications where the change in capacitance was measured with respect to the change in relative humidity. The relative humidity was varied between 20% and 92%.

**Figure 11:**
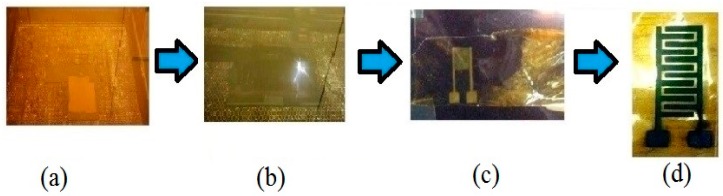
Fabrication of the graphene-PI based sensor. (**a**) The PI film was attached to the glass substrate, (**b**) laser writing on the polymer film to develop the electrodes, (**c**) the laser-induced graphene was transferred to Kapton tapes, (**d**) the final sensor patch after the fabrication process. Reproduced from Nag, A., Mukhopadhyay, S.C. and Kosel, J., 2017. Sensing system for salinity testing using laser-induced graphene sensors. Sensors and Actuators A: Physical, 264, pp. 107–116 [99].

Nag et al. examined the generation of laser-induced graphene from polymer films [99]. This was based on the production of conductive material from commercial polymer films [100]. The high electrical conductivity of the electrodes (~104 S/m) and the high flexibility of the substrate, with a bending diameter of 6 mm, were two of the significant advantages of this sensor patch. Figure 11 shows the fabrication steps. Low-cost polymer films were attached to a glass substrate and the electrodes were patterned by the laser. The laser parameters associated with this fabrication process were 9 W, 70 m/min, and 1 mm for power, speed, and z-axis, respectively. The graphene was then transferred to Kapton tapes to use the formed material as electrodes. The formed graphene was transferred onto Kapton by placing the Kapton tapes over the conductive material and manually pressing it. There were two reasons why the laser writing was done separately on the PI films and not on the Kapton tapes. Firstly, due to the sticky nature of the tapes the induced graphene would have coagulated, thus tampering the design. Secondly, photo-thermal induction could not have taken place on the Kapton tapes (1000 microns) due to its greater thickness in comparison to the PI films (120 microns). The transfer was done carefully to preserve the design of the electrodes. The difference in conductivity between the induced and transferred graphene was less than 200 mS/m. The final sensor patch consisted of six pairs of interdigitated electrode fingers, each having a length and width of 500 microns and 100 microns, respectively. Based on the electrochemical characteristics of the sensors, they were then used for different environmental applications such as different concentrations of salinity, nitrate [101] in a range of water bodies, and taste-sensing applications [102]. The change in the impedances values including resistance and reactive values were determined in accordance to the changes caused by the input values.

**Figure 12:**
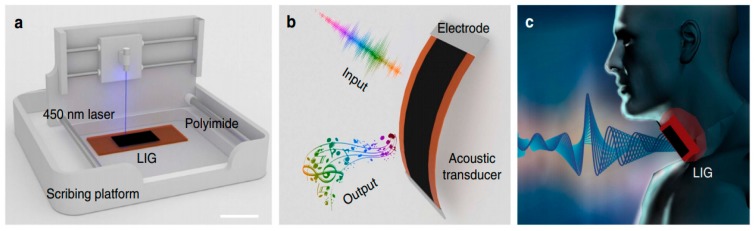
Schematic representation of the manufacturing procedure and the morphology of the LIG sensor. (**a**) The one-step manufacturing process of LIG, (**b**) LIG can produce and sense sound in one device, (**c**) the artificial throat can sense the movement of the throat and produce manageable sound. Reproduced from Tao, L. Q., Tian, H., Liu, Y., Ju, Z. Y., Pang, Y., Chen, Y. Q., Wang, D. Y., Tian, X. G., Yan, J. C., Deng, N. Q. and Yang, Y., 2017. An intelligent artificial throat with sound-sensing ability based on laser-induced graphene. *Nature communications*, *8*, p.14579 [105].

Yoo et al. reported a laser-induced direct graphene patterning and simultaneous transferring technique for a graphene sensor platform [103]. In this study, a laser-induced pattern transfer (LIPT) technique was proposed for transferring and patterning graphene via a single-step procedure. The significance of this work is related to the reduced complexity of the fabrication of graphene that is attached with conventional techniques like CVD and Hummer’s method. Another attribute of this work lies in the smaller duration of the of pulse of the femtosecond laser in comparison to the duration taken by the photons and electrons to transfer energy between them. This creates an advantage of completion of the laser-ablation process in a very short time, thus causing minimal lateral thermal damage on the substrates. One of the important steps related to the fabrication of these sensors is the proper attachment of graphene on PMMA layer due to the variance in the surface tension created between graphene and the PMMA layer. The patterning of the suspended graphene/PMMA layers was done to form the holes using a template, which was taken off to form the patterns. The laser parameters for power and wavelength were kept fixed at 8 nW and 402 nm, respectively, to generate a cyclic response of the change in the relative current towards a bias voltage fixed at 2.5 mV. A similar work to [99] was shown by Tao et al. [104], where laser-induced graphene was also generated to form flexible sensors. However, this work had an additional advantage of having a sound-sensing capability, which could be used as an intelligent artificial throat [105]. As shown in Figure 12, the artificial throat has the integrated functions of producing and sensing sounds. When an AC potential was applied to the sensor, the periodic joule heat originated the development of air, resulting in sound waves. When a low voltage was provided to the device, the vibration of throat cords changed the sensor’s resistance, causing the fluctuation of the current. Hence, the sensor can work as a sound source and sensor at the same time. The operating principle of the LIG artificial throat is shown in Figure 12c.

### 2.3. 3D-Printed Sensors 

The third type of printed-sensor prototype is based on 3D-printed sensors. Most of these sensors were fabricated using laser-sintering or laser-tapping techniques on the flexible substrates. 

Watanabe et al. [106] used copper nanoparticle ink and fabricated a copper interdigital electrode by laser direct written copper micro-pattern. This copper micro-pattern of the electrode was printed on a flexible polymer substrate of polyethylene naphthalate (PEN) using the 405-nm laser beam. A graphene oxide was coated on the copper electrode for using the device as a humidity sensor. Since the graphene oxide has a good hydrophilicity and high oxygen content, the device works perfectly for humidity-sensing. The disadvantage of this flexible sensor is that the flexibility of the polymer substrate may be damaged due to its repeated heating during sintering of the micro-pattern of the electrode. 

**Figure 13:**
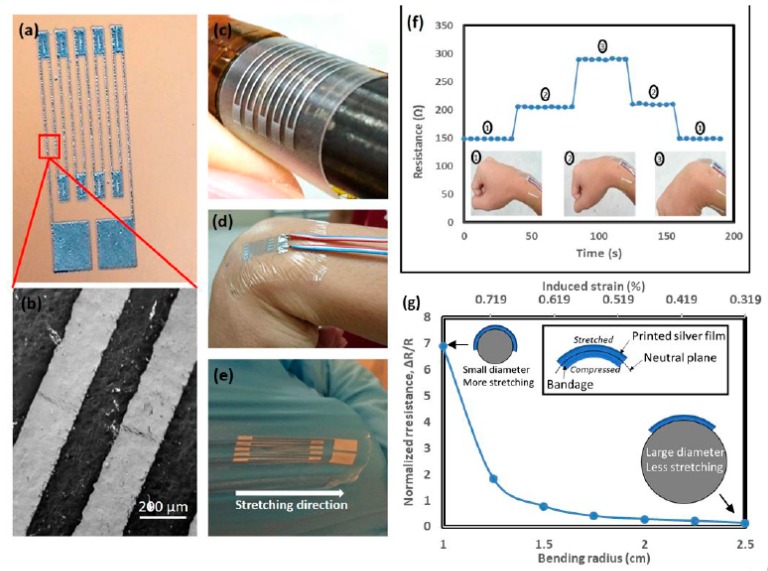
(**a**) The aerosol jet-printed strain sensor consisting of bandage as the substrate and (**b**) silver nanoparticles as electrodes in the form of electrical tracks. The sensors had (**c**) a small bending radius of curvature that assisted them to be stuck to (**d**) a human wrist. (**e**) The responses of the sensors were highly repeatable when they were attached to (**f**) the elbow, to analyze their bending. (**g**) The change in relative resistance with respect to the bending radius [62].

Agarwala et al. [62] developed a wearable strain-sensor based on silver nanoparticles on a low-cost polymer substrate using laser-sintering for home-healthcare applications. The sensor was developed at a laser power of 120 mW and scanning speed of 70 mm/s by six scan passes. This strain-sensor was integrated on a commercial bandage due its high stretchability. It could be used by rolling around the wrist due to its flexibility and the strain caused by human joint movements could be measured easily. This research group noticed that the sensor was able to provide good sensitivity and a stable response for 700 cycles of repeated bending. At no strain, the resistance of the sensor is 150 Ω; when the wrist is bent 45˚, the resistance was 200 Ω. Figure 13 shows the developed sensor consisting of printed electrical tracks on bandages and their responses towards elbow bending with respect to the radius of curvature. 

Huang et al. [107] fabricated a paper-based multilayer circuit using electricity-conducted silver nanowires (Ag-NWs) by laser-printing technology, since the electrical property can be controlled by the amount of silver nanowire. The content of this nanowire printed on the flexible polyimide (PI) film is the main factor of the resistivity of the circuit. The temperature is also an important factor because the adhesion property of the material depends on it. This research group tested and concluded that, at the temperature of 180 ˚C, the circuit showed a good response. Above this temperature, the silicon rollers of the circuit may get damaged. This is why the scanning speed of the laser had been selected at 2 m/min and the heating time of the substrate was ~1–2 s. One of the main advantages of this paper based circuit is that the paper does not get wet during the fabrication process. Also, more than one printing can be done in the laser-printing process for a multilayer conductive bridge. Thus, the performance optimization can be controlled in electronic applications.

Rahimi et al. [108] fabricated a highly stretchable strain-sensor by partially-aligned graphene and carbon nanotubes particles on a polyimide substrate that can be used to detect the joint-bending motion. A CO_2_ laser was used to print the carbon patterns on a polyimide film so that the carbon flakes have the same direction of the scanning path. The main advantage is that the strain-sensor is highly sensible and the gauge factor is around 20,000. Due to its unidirectional sensitivity, the gap between the conductive particles increases during longitudinal strain and, as a consequence, the resistance increases. The conductivity of the carbon patterns depends upon the input laser parameters, like laser power and scanning speed.

**Figure 14:**
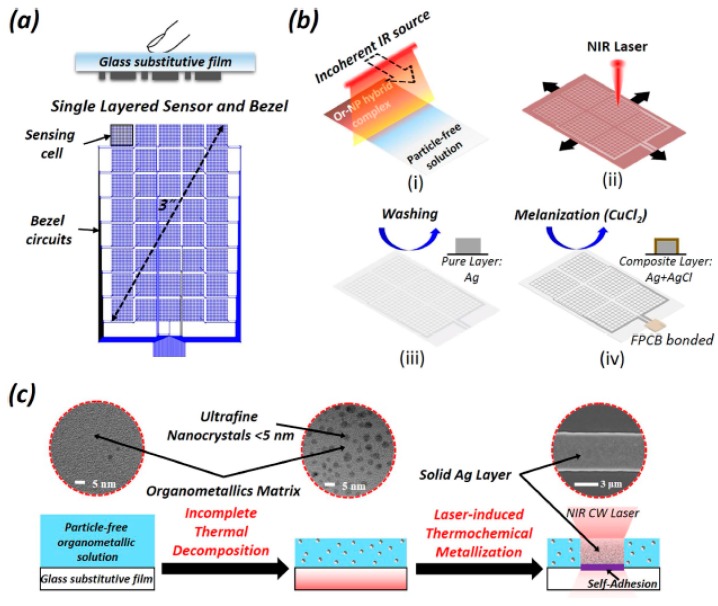
The (**a**) cross-sectional view and (**b**) schematic diagram of the fabrication steps of the developed touch device. The different steps included the (i) in situ generation, (ii) laser-processing (iii) rinsing and (iv) melanization processes. (**c**) Schematics of laser induced microfabrication using thermochemical direct metallization done on enhanced organometallic/ nanoparticle hybrid complex during the laser-induction process to form particle-free organometallic solution. The inset shows the transmission electron microscopic image of the hybrid complex and scanning electron microscopic (SEM) image of the laser-processed layer on the left and right, respectively [109].

The fabrication of a single-layer, flexible touch-sensor was shown by Son et al. [109], exhibiting their usability as wearable devices, deterring the heterochromia, and interference problems. The significance of this work lies in the novelty in the entire processing of materials, structuring, and fabrication of the sensors. The sensors were formed using indium-tin-oxide, one of the materials which has combined advantages of high conductivity, better chemical stability, and high resistance towards change in its responses toward environmental changes. Another highlight of this work can be described as the development of single-layered bendable screens without the need of any conventional techniques, like photolithography. These sensors showed a new direction toward the use of polymers in the electronic world. A polymeric film 500 μm thick was used as the substrate for the sensors, which was processed with laser-induction technology at a temperature below 150 ˚C. The substrates were then spin-coated with an organometallic silver precursor before laser processing. The samples were then processed with a laser with a continuous wave of power and wavelength of 10 W and 107 nm, respectively. The use of a continuous wave instead of a pulsed wave gave the fabrication process an increased sensitivity and high speed in its completion. The entire laser process was completed via sub-dividing it into two sub-steps, involving the fabrication of the array of sensors and then subsequently connecting the bezel circuits for the transmission of electrical signals. Due to its main advantages of outstanding mechanical and optical properties, it can replace the conventional multi-layer touch screen panel as it has a unique sensing capability of deviation of capacitance by separate sensing pixels. The resistance of the sensors also changed symmetrically with respect to the loading cycles for different bending curvatures. Figure 14 represents the schematic diagram of the fabrication steps, including in situ generation, laser processing, melanization, and metalization processes [109]. 

Zhao et al. [110] showed the use of pulsed laser-sintering technique to print a nano-alloy ink on a PET substrate. The significance of this work lies in the conjugation of plasmonic coupling of nano-alloys and pulsed-laser energy. Another advantage of these sensors lies in the development of the conductive patterns with an alloy including gold, which increases the stability of the electrodes. An alloy consisting of copper and gold nanoparticles was synthesized at a low temperature with controlled size and composition. The heat generated from the pulsed-laser-sintering method caused a localized heating of the nanoparticles. This nano-scaled heating affected the size and composition of the nanoparticles, thus affecting their electrical and thermal properties. The power, frequency, pulse width, and speed of the laser nozzle during the sintering process was 0.5 W, 100 kHz, 30 ns, and 70 mm/s, respectively. The sintering effect of chromium was also analyzed, along with gold, on the PET substrates, which showed that there were minute differences in their binding energies which was caused due to the corresponding difference in their activation energies. These differences were caused as a result of difference in the nanoparticle sizes and their surface velocities on the substrates. The sensors were employed for the detection of volatile organic compounds, which have significant importance as wearable-sensors for environmental sensing. A change in the relative resistance was observed when they were tested for volatile compounds in vapor concentrations in ppm levels. This change resulted due to the corresponding changes in the inter-particle distance and dielectric properties, thus resulting in the change in electrical conductivity. Luong et al. [111] developed a 3D-complex graphene foam by laser printing process that can be useful for electronic sensor applications. After developing the object by the LOM process, a 50-W fiber laser was used to shape the object to a desired internal geometry. This research group developed an arterial-pulse sensor which can record the artificial pulses when the sensor is wrapped around the wrist. This 5-cm long strap gives an output by sensing the blood flow. In the next application scenario, pulse waveform amplitude of the sensor can be used to measure the blood pressure. 

Renn et al. [112] analyzed the experimental results from the different parameters of the laser-sintering method for fabricating a flexible sensor. They have shown that the localized heat affected zone (HAZ) should be minimized for printing the metal on a flexible substrate, unless higher temperatures can damage the underlying substrate. Since the metal can be oxidized easily by the environmental oxygen, the printing process can be done in an inert atmosphere, unless it will be difficult to get the proper material properties of oxygen-sensitive nanoparticles, like nickel and copper alloy. The experimental analysis of this research group helped to find out the advantages of shroud-gas-shielded laser-sintering of copper and copper–nickel alloy inks and strain gauge. 

## 3. Current Challenges and Future Opportunities

Even though an enormous amount of work has been done on printed flexible sensors, a lot of issues still need to be addressed in the current scenario. The characteristics of nanocomposite-based flexible sensors are primarily degraded due to the formation of the composites. The electrical conductivity of the nanocomposites is largely deteriorating due to the inclusion of the polymer matrix. This subsequently affects the performance of the sensors regarding their reliability and sensitivity. The compatibility issue of the materials mixed to form nanocomposites is also an issue that needs to be studied. Some of the materials need to be processed before they can form composites, which increases production time and cost. Another disadvantage of nanocomposite-based sensors is related to the inability to optimize all the sensing parameters. For example, while increasing the sensitivity of the sensors, certain attributes like thermodynamic stability becomes worse in SnO2-CuO-based sensors [113]. Some other issues for nanocomposite-based printed sensors that need attention are the non-uniform distribution of the nano-fillers, formation of agglomeration, and non-uniform cutting of the electrodes [114]. Furthermore, there are compatibility issues with the sensors when they are used for healthcare or environmental applications; in certain cases, the biocompatibility of the sensors makes them unsuitable for wearable ubiquitous sensing purposes. The performances of nanocomposite-based sensors are also affected when they are coated with additive layering for selective recognition of analyte molecules. The sensor-structuring of electrodes curved from nanocomposites also needs to be researched further, especially for non-aligned nano-fillers. For laser-induced printed sensors, the change in the properties of the ablated materials is a topic that merits further consideration. Even though researchers have worked with different kinds of lasers for laser-induction, the by-products after the ablation process are sometimes toxic. The ablation of polymeric substrates also makes them difficult to form composites due to the thermal effects from the laser. The contribution of the laser parameters on the finished products also needs more analysis to form sensors with better performances. The 3D-printed mold-based techniques also have certain demerits, like high energy consumption, too much dependency on thermoplastics, emission of dangerous carcinogenic particles, and the limited potentiality of casting polymers and conductive materials. The printed molds have to go through certain cleaning processes before they can be used as templates to form sensors. The accuracy of the dimensions of the molds is another area that researchers should focus on. In comparison to other fabrication techniques, like photolithography, the 3D-printed molds are not accurate enough. Also, only a limited number of 3D-printers employed across the world can print more than one material at a time. This limits the versatility in the fabrication of the sensors. The above-mentioned problems should be worked on to optimize the use of printing technology for producing sensors with better quality. 

Apart from the previously mentioned challenges about the raw materials, there are still some issues that needs to be fixed related to fabrication techniques. For nanocomposite-based laser-ablation, it can be an issue to precisely ablate the top layer to form the electrodes of the sensor. This is mainly because the heat generated by the laser modulates the polymeric substrate beneath the conductive layer, which alters the mechanical characteristics of the sensors. One of the remedies for this issue could be to create a multi-layered structure, where the conductive layer would affect the base substrate. Another solution may be the development of a singular layer of the sensor, which consists of both substrate and electrodes; this way, the ablation of part of the prototype will not affect the other parts. One of the problems faced by the laser-ablation of the samples in terms of the sintering process is the effect of characteristics of the electrodes or substrates that determine the characteristics of the sensors. Also, in the sintering technique, apart from with some polymers like polyimide, most polymers have not been worked upon. The conjugation of two materials in the laser-ablation process is another issue which can be resolved or further improved by enhancing the sintering process on different materials. For 3D-printed sensors, the biocompatibility of the developed prototypes for the chosen application is an issue researchers are currently working on [115]. The usability of the sensors for a specific application largely depends on the characteristics of printing filament. The sensors can have negative side effects because of these printing filaments, especially if they are used as implantable prototypes. Work should be done to develop biocompatible filaments that can be used to develop sensors for all purposes. Some of the other disadvantages of the currently available 3D-printing machines are their high energy consumption, cost-ineffectiveness, and emission of harmful gases. This can be dealt with by developing eco-friendlier printing machines with better technologies that operate on a wide range of printing filaments. The conjugation of wireless protocols, like Bluetooth or Wi-Fi operating on a power saving mode, is one way to reduce the input power requirement.

**Figure 15:**
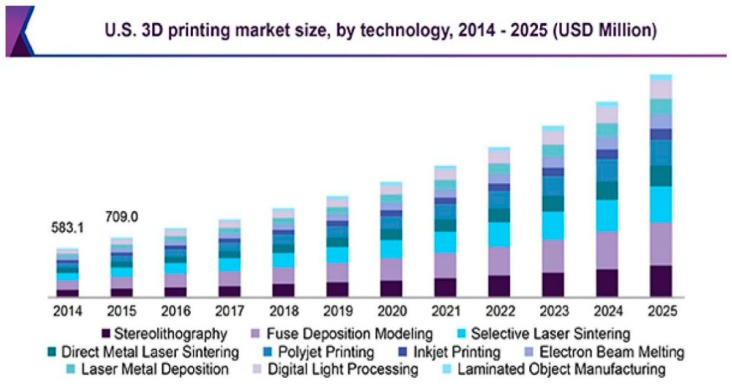
Market survey of the increase in the printing technology for US market for years from 2014 to 2025. Reproduced from 3D-printing market size, share, and trends analysis report. https://www.grandviewresearch.com/industry-analysis/3d-printing-industry-analysis [119].

The market trend of the printed electronics is growing every year [116]. Between 2017 and 2027, most printed sensors were used for tactile, temperature, pressure, gas, image, and bio-sensing purposes [117]. The sensors to be developed further are predicted to be fabricated using different kinds of printing technology, based on the processed materials, dimensions of the final products, and proposed applications. Among the processing materials, some of the substrates that are being considered are glass, silicon, paper, plastics, PET, PEN, and PI. Some of the sectors that these sensors will be considered for implementation in include wearable sensing, automation, military, robotics, and environmental monitoring. A few of the attributes of the printed sensors for the upcoming years are their low cost of fabrication, eco-friendly nature, and innovative sensing prototypes [118]. Figure 15 shows the market survey for the increase in the 3D printing technique for the US market during 2014–2025 [119]. It is seen that a wide range of printing techniques has been considered to form these sensors. The rise in the cost every year also includes an increase in the number of sensors used for monitoring purposes. It can be concluded from the given trends that an increase in the use of printing technology would assist in employing the developed sensors in different sectors.

## 4. Conclusions

The paper provided a substantial review of some of the significant research works done on the fabrication of printed flexible sensors. The specificity of all these sensors is the assistance of the utilization of lasers for processing the materials to form the sensing prototypes. The laser processing has been divided into three categories, depending on the type of materials being processed. Processing had been done on all these forms by varying the laser parameters like power, speed, wavelength, etc. The printed flexible sensors were divided into three categories, namely nanocomposite-based, laser-induced, and 3D-printing techniques. The nanocomposite-based techniques compromised of laser-ablation on the different kinds of nanocomposites developed from carbon materials, metal-oxides, and polymeric substrates. The second category includes the laser-processing or ablation on a range of substrates to form or modify conductive materials of the sensing prototypes. The third category includes the 3D-printed sensors, where laser-tapping or laser-sintering had been done on the raw materials to fabricate them. The differences in these processes create sensors with a wide range of electrical, mechanical, and thermal attributes. The employment of printing technology has specific advantages associated with them, making them a favorable choice for the fabrication-sensing prototypes. The laser-cutting techniques illustrated above benefit from a low cost of fabrication, quick sample preparation, and enhanced sensor characteristics. Three different types of techniques offer unique properties to provide optimal performance in different applications. The dynamic nature of these sensors allows them to be used in the healthcare, industrial, and environmental sectors. Even though a lot of work has been done related to laser-assisted printed sensors, there are still some loopholes, as mentioned above, to be closed for further enhancement of printable flexible electronics. 

## Figures and Tables

**Table 1:** Comparison of the performance of the sensors developed from the three types of laser-assisted printing technology.

Materials	Type	Sensor Type	Processing Parameters	Res. Time (s)	Application	Ref.
MWCNTs/PDMS	Nanocomposite-based	Cap.	P:24 W, S: 70 m/min, FD: 1 mm	1	Limb movements, respiration	[1]
Silver/PDMS	Nanocomposite-based	Res.	P:120 mW, S: 2 m/min, FD: 1 mm	~5	Pressure sensing: 50-100 kPa	[57]
Polymer/carbon black	Nanocomposite-based	Res.	P: 150-300 mJ/cm^2^, Pulse duration: 30 ns, FD: 3 cm, F.: 10/20 Hz	1.2	Toluene vapor sensing	[58]
Titanium dioxide/Ag	Nanocomposite-based	Res.	Nd:YAG: beam wav.:1064 nm, W: 532 nm, repetition rate: 6 Hz, PD: 10ns	0.2	Gas-sensing: NO_2_: 60 ppm, NH_3_: 200 ppm	[59]
Graphene/Polyimide	Laser ablation	Cap.	P: 10%, S: 25mm/s,	40	Gas-sensing: CH_4_, H_2_O, CO_2_, CO: 10–100 ppm	[60]
Aluminium/PET	Laser ablation	Cap.	P: 24 W, S: 70 m/min, FD: 1 mm	1	Tactile-sensing: Pressure:	[2]
Graphene/Polyimide	Laser ablation	Res.	W: 10.6 μm, FD: 5 cm	214	Gas-sensing: NH_3_: 75–400 ppm	[61]
Silver/PU coated with a layer of acrylic	3D printed	Res.	P: 120 mW, S: 10-150 mm/s, W: 780 nm, FD: 60 µm	492.16	Strain-sensing: 10–30 MPa, 7 N	[62]
Silicon elastomer	3D printed	Optical	Energy: 2.5 mJ, F: 250 Hz, EX: ~1 min, length: 3 mm, SB: 0.21 nm	–	Tactile-sensing: Different loads from 50–250 g	[63]
Graphene/PBS	3D printed	Res.	P: 1mW, SR: 2.5 cm^-1^, W: 514.5 nm	1–27	Gas-sensing: Methanol, Toluene, Hexane, 1,4- Dioxane, Diethyl ether, Dimethyl carbonate	[64]

* P: Power; S: Speed; W: Wavelength; PD: Pulse duration; FD: Focal distance; F: Frequency; Res: Resistive; Cap: Capacitive; SB: Spectral bandwidth; EX: Exposure time; PBS: Polyborosiloxane.
